# Book Reviews

**Published:** 1832-04

**Authors:** 


### CRITICAL ANALYSES.

Quæ laudanda forent, et quæ culpanda, vicissim,

Illa prius, cretâ; mox hæc, carbone, *notamus.*—Persius.

*Observations on the Medical Treatment of Insanity.* By Edward J. Seymour, m.d., Physician to St. George's Hospital, Consulting Physician to the Seaman's Hospital, and Physician to his Royal Highness the Duke of Sussex.—8vo. pp. 95. Longman and Co., London.

This little volume contains the substance of the Croonian lectures delivered by Dr. Seymour, before the College of Physicians, in May 1831; and we feel justified in saying, that we have rarely found in so small a compass, so much instructive matter, or so many sound practical observations, upon the very important subject of the medical treatment of insanity. The learned author has not drawn only upon his own resources; he has consulted the works of many eminent continental writers, and especially that of Dr. Guistain, of Ghent, on Maniacal Diseases. We fear it is too true that "the hopeless nature of many cases of insanity, the severity of others, and perhaps the necessity for seclusion, have induced physicians very generally to resign all hopes of applying, successfully, means of cure, which may be said, in other diseases, to give all but life itself." The remarks made by Dr. Seymour will tend much to strengthen the confidence of the physician who has to treat disorders of the mind. He shews that, under judicious care and treatment, persons recover from even the most extreme instances of aberration, and in such a proportion as ought to stimulate practitioners, so far from abandoning their patients, to use every resource resulting from their observation and derived from their art.

"To exemplify my meaning, I may take the cures in Mr. Warburton's establishment, the White house on Bethnal Green; a house where the arrangement, order, and attention, are highly deserving of praise, and which contains four hundred lunatics, comprising persons in every stage and variety of this afflicting malady. It is to be observed that many of these cases are paupers, received after a probationary sojourn in the parish infirmary, not unfrequently dismissed uncured, after the year's trial, from Bethlehem or St.

Luke's. In the year 1829, of two hundred persons admitted into the White house, fifty were discharged cured in the course of the same year; and, creditable as such a fact is to the establishment, it is still more important to medical men, as one which may rouse them to use all their energies in the treatment of a disease which may and can be cured." (P. 3.)

Even in the most extreme cases, cures have been effected by diligent and judicious management and medical treatment, and of this fact the following proof is given. In such instances so happy a result may be rare, but the success which has been once obtained under such formidable circumstances may be obtained again; and the physician will not perform his duty if he, from apathy or distrust in the power of his art, consign his patient to a life of madness, without having used every possible effort for his relief.

"The following instance is related by Mr. Finch, a medical man, and the proprietor of a house for lunatics, at Laverstock, near Salisbury, in his evidence before the Committee of the House of Commons, in 1816: 'I can give you (says Mr. Finch) a very strong case of a patient I had from St. Luke's; he was a man of opulence, sent there as a pauper; he came to me afterwards a gentleman. This man came to me a most miserable object from St. Luke's, after having been there a twelvemonth, and discharged as incurable: he was so bad that he had lost nearly the use of his limbs: he walked upon his toes; he could scarcely get from the coach to my house; the muscles of his legs were contracted; he was so nasty in himself that he ate his own fæces, and would his own flesh, if he had not been prevented; he tore it immediately, as he came to me. I had to put him into a room where he could do no mischief to himself or any one else, but took off every restraint: I found him within a few days more composed. Some little time afterwards he became so bad again, that I was obliged to use some restraint, so as to prevent his eating his own fæces. From having a man attending him two or three times every day to the privy, his disposition to filth was lessened; by attending to his bowels, and keeping him strongly exercised in the gardens and fields, I found him gaining strength daily; within six weeks, capable of playing at bowls; and I sent him home perfectly restored in four months, where he carried on the business of a coach-proprietor for three years afterwards." (P. 4.)

It is essentially necessary also that the practitioner should not hastily conclude, as we fear he too frequently does, that, if a person has been insane for a considerable length of time, his recovery cannot be hoped for. Time is not conclusive: "solitary instances of cure have occurred after uninterrupted insanity of ten years."

Insanity may spring from physical or moral causes. This division has been long admitted by attentive observers, and it is of great practical importance.

Dr. Seymour first investigates the alterations of structure which have been noticed by authors whose great opportunities of observation entitle them to confidence, as occurring on opening the bodies of lunatics.

"Nothing probably is more difficult than to estimate the exact worth of such alterations in structure. Every one who has studied morbid anatomy must have been struck with the amazing extent to which disorganization of the brain may be carried, without the intellectual functions being impaired. Great and intense pain exists in the brain for months; it is occasionally accompanied by vomiting, followed by diminution of the perfection of the external senses; there is dimness of sight or deafness; the smell is morbidly acute; paralysis follows. On opening the brain tumors are found, either scrofulous or malignant, in its substance; yet, from the commencement to the conclusion of the disease, the mind has never varied, the patient has not suffered in perception or volition, her affection for her family has been unimpaired, she has been capable of consulting on affairs of importance, and able to enter into and receive comfort from the highest duties of religion: and this is no hypothetical case. Is it not bold, therefore, to assert, that where none of these signs of physical infirmity exist, where there is neither depraved sense, nor diminished motion nor sensation, but where a train of incoherent ideas, shuddering fear, or extravagant ecstasy, or gloomy pride, are present, that it is useless to apply to the art of medicine, for these probably depend on organic disease? Even where, after death, slight deviation from the ordinary appearance of the brain or its vessels is found, it is very necessary to be cautious in forming an opinion; for similar appearances exist in cases which have never shewn symptoms of aberration of intellect." (P. 8.)

Dr. Seymour first refers to the experience of Dr. Haslam. In the cases related by Dr. H., the appearances described after death are by no means convincing of the physical nature of the disease. Dr. S. remarks, that persons who carefully observe dissections of patients dying of different diseases, will be struck by the variety of thickness or thinness in the bones of the skull, and the variation in the degree of firmness or softness of the brain, dependent on the season, the length of time during which the patient has been dying, and on age; and such great variations occurring in cases in which no alteration of perception existed during the life of the patient.

"The same may be said of the presence of vesicles in the choroid plexus: such bodies being found occasionally in maniacal cases, by no means proves that the malady owed its origin to this deviation from natural structure, since they exist very frequently, and to a great extent, without the functions of the brain apparently suffering. There is, perhaps, however, no appearance on which so much stress has been laid as on congestion of the vessels of the head; the mechanical physician having his scruples satisfied by a very slight injection of the vessels of the brain, while the morbid anatomist must have observed almost every variety of intensity of this appearance, often unattended by corresponding symptoms of excitement, or disturbance of the brain, during life. Of effusions into the ventricles of the brain, the same may be said: fluid is undoubtedly present in a healthy condition, as may be, and has been, proved on dissection of living animals, and it probably varies in different animals, according as their life is more perfect; large, strong, and lively animals having a larger proportion than those in a weak or pining condition. It has repeatedly occurred to me to witness a few drachms of fluid in the ventricles assigned as the cause of death, when I have immediately afterwards observed, in the dead-house of the hospital, twice the quantity, in cases where the patients had died of inflammation of the viscera of the abdomen, or some other disease, with which, visibly at least, the functions of the brain had not sympathized during life. It is remarkable that, in that particular alteration of the structure of the brain which principally occurs in aged people, and to which the French pathologists have given the name of *ramollisement de cerveau*, in the numerous cases related by M. Rostan, almost all of which occurred in persons above fifty years of age, notwithstanding the frequent alteration of sensation and motion, and the still more frequent change in the external senses, the intellectual faculties often remained unchanged." (P. 10.)

In this country physicians generally incline to the belief that congestion of the brain is the immediate cause of insanity, or thickening of the membranes, especially of the arachnoid membrane, or preternatural thickness of the bones of the skull: others, on the continent more especially, have been more precise, one author having frequently observed calcareous matter in the pineal gland. Meckel speaks of the preternatural hardness of the corpora striata in cases of melancholy. Gall considers that the crura cerebri and the corpus callosum are principally affected in suicides, and that this form of madness is chiefly accompanied by thickness of the skull, and its increased weight. From these considerations it would be fair, Dr. Seymour believes, to conclude that, by calm and extensive observation, the number of cases of mental derangement from organic disease of the brain will be greatly diminished; and such, in fact, is found to be the case. Upon this point, the experience of Esquirol is referred to: he relates that, on inspecting the bodies of 168 melancholic patients, two had the brain harder than natural; four, organic disease of the substance of the brain; three, where cartilaginous bodies were found upon the falx; and five, where extravasation of blood had taken place into the cavities, and in the substance of the brain. But although the disturbance of the functions of the sensorium constitutes the disease, this disturbance may arise from that inexplicable connexion with distant parts which, for want of a clearer expression, has been termed sympathy. "Hence physicians have sometimes been led to seek for the material cause of insanity in the viscera of the thorax or abdomen; the brain sympathizing with the injured part, and its perceptions being altered."

We are convinced, with Dr. Seymour, that the mutual sympathy between the heart and brain is little understood, at least by the generality of practitioners; but it must be confessed that much light has recently been thrown upon this topic by different writers.

"Acute diseases of the brain will occasionally arise, and run their course, with little or no perceptible disorder of the circulating system; while, on the other hand, repeated inflammations will attack and disorganize the heart, without the functions of the brain being in any way impaired. There are, however, great exceptions to such observations. The commencement of acute carditis is often very obscure, and occasionally the first and most violent symptom has been maniacal delirium. It has occurred to me recently to see two cases in which the sympathy of the brain with the injured organ was very obvious: the first was that of a young gentleman who had suffered from repeated attacks of rheumatic pericarditis, during the last of which his memory became impaired, his ideas were uniformly of the most melancholy nature, phantoms presented themselves to his imagination, and his mind was a prey to terror. The patient died, and the heart, as was expected, was found to be extensively disorganized, by the enlargement of its muscular parietes, and the deposition of repeated layers of lymph in the pericardium. Another case occurred in St. George's Hospital, under my care. The patient had suffered from acute rheumatism, and the heart had been attacked by rheumatic inflammation. About the period when he appeared to be convalescent, suddenly violent maniacal symptoms arose, attended with a prevailing fear that he was doomed to expiate, in prolonged tortures, crimes probably imaginary. Local depletion in the region of the heart, and the constant application of ice to the head, during many successive days, removed the symptoms, and the patient has been now during several months quite well.

"Corvisart, in his beautiful work on diseases of the heart, adverts to the influence of the heart on the intellect, remarking that in the third period of aneurism of the heart, the patient is often attacked with furious delirium. It is probable, likewise, that some of the cases of insanity arising from perverted passions, disappointed affection, loss of friends, sudden diminution of worldly possession, or the spectacle of some unusual and appalling crime, may be intimately connected with the influence of such impressions on the circulation. It is notorious that, during the terrible scenes of the French revolution, organic diseases of the heart, as well as cases of aberration of mind, were wonderfully increased; and it is far from improbable that the diseases of the heart, brought on by long watching, frequent peril, and urgent anxiety, were productive, in their turn, of such an influence on the circulation in the brain as to alter its powers of perception. In advanced life, there is no doubt that the influence of the diseased structure of the heart is the principal cause of the most frequent and most fatal of the diseases of the brain. Sanguineous apoplexy, if not in all, certainly in the majority of cases, arises from the increased size and force of the muscular parietes of the left side of the heart, impelling blood into the vessels of the brain, which vessels are rendered weak from steatomatous, or unyielding from bony deposits in their coats. In studying, then, the organic changes in the human body which produce alteration of the functions of the brain, it will not be sufficient to observe the condition of the brain alone, the state of the heart and great vessels must be carefully investigated; and these being found healthy, we still are not enabled to conclude that no local cause exists which may have excited sympathetic disturbance in the sensorium." (P. 13.)

A third series of organic causes is to be found in the diseases of the abdominal viscera, between which and the functions of the brain there is a wonderful sympathy. It is true that inflammation of the bowels will proceed rapidly, and terminate fatally, without involving sympathetically the intellectual functions; "but, on the other hand, the degree in which these functions, and even the external senses, are disturbed, by the diseased secretions of the stomach, liver, and small intestines, is manifest to every one. The severe and agonizing headach experienced by some patients, from the simple distention of the great intestines, is a familiar instance of this connexion." Hence our attention is carried to the condition of the viscera of the abdomen, and great care has been taken by continental observers in investigating this condition. From their researches it appears that certain peculiar appearances in the great intestine had struck, simultaneously, physicians in different countries, as presenting themselves in the bodies of melancholics. Greding, and other German physicians, noticed this change; but it was first made public by Esquirol, in his memoir on the subject of the Displacement of the Colon in Madness.

"Mons. Esquirol relates numerous cases in which the arch of the colon was found after death displaced; often low down in the pelvis, and always in the hypogastric region. He expresses himself in the following words on the alteration in position:

"This displacement cannot be attributed to a mechanical cause, dependent on the thickening of the parietes of the colon, or the accumulation of fæcal matter in its interior, for, in the greater number of subjects which I have opened, the colon was empty, and in all healthy. The same was the case with the ascending and descending portions of the colon, which by their action might have dragged down the transverse portion. Neither is this displacement the effect of the last disease of the patient, for the same appearance presented itself in the bodies of maniacs who died of different diseases." (P. 16.)

It should be remembered that, in the majority of cases related by Esquirol, where these appearances presented themselves, organic disease of the brain also existed. Dr. Seymour is not aware that this peculiar change in the great bowels has been observed in this country, either at Bethlem or elsewhere. Esquirol states, that, in melancholia, this alteration was found in thirty-three out of 168 patients. In reference to this subject, Dr. Burrowes* observes, that Morgagni and others have remarked these varieties in the colon, but not as peculiar to insane persons. The most evident causes for the disturbance of the functions of the brain, from sympathy with the disturbed functions of other parts, are found in the various changes which occur in the generative system; and upon this subject Dr. Seymour makes some good comments.

Among the physical causes of mania, the introduction of poisons into the constitution is enumerated by all authors; the three most remarkable are alcohol, opium, and mercury. By the improper use of ardent spirits, "delirium tremens" may be produced, or even furious mania. It is remarkable what enormous quantities of spirits may be taken without injury either to the mental or bodily health. Dr. Seymour knew a patient who for many weeks drank a quart of undiluted spirit (gin) daily, often a pint before breakfast, and yet this man was entirely free from bodily ailment. Similar facts have fallen under our own observation.

The use of opium, carried to a great extent, appears to produce a similar effect to that of alcohol; exalting the sensibility of the brain, until, by long perseverance, exhaustion ensues.

"It occurred to me, when learning my profession at Edinburgh, to see a very remarkable instance of delirium tremens, from over indulgence in the use of opium. A tradesman, in a respectable condition of life, finding his business decline, took refuge from care in the delightful sensations produced by opium; by degrees he increased the quantity, until the dose amounted to two drachms of solid opium; at length, from distress, he was reduced to extreme poverty, and unable to purchase a temporary solace for his misery. In this state he was brought into the infirmary; his mind was disordered, and the bed shook under him from the violent and involuntary trembling of his limbs: a dose of opium relieved him, but the instant the effect wore off the same wretched state of mind and body returned. The cure was effected by gradually diminishing the quantity of opium at each dose, and by carefully preventing his leaving the hospital. After some months he recovered; the humanity of his physician obtained him employment in his former business, and he gave his word (and happily had the fortitude to keep it) never again to take opium in any form. It occurred to me to know, two years after that time, that he was in a thriving and happy condition." (P. 27.)

To another set of causes of insanity, to which all writers attach considerable weight, Dr. Seymour briefly but forcibly refers; viz.

* Commentaries on Insanity, p. 89.

certain depraved habits in both sexes. Upon this subject the essays of Dr. Beddoes, entitled "Hygeia," are replete with information which demands the especial attention of the heads of large schools for either sex.

Dr. Seymour next proceeds to describe the remarkable symptoms of mania arising from moral causes, and to point out some of the difficulties of diagnosis. As we are anxious to bestow as much space as possible upon the analysis of the last lecture, we cannot enter upon these topics, interesting as they are in themselves, and ably treated by our author.

We pass on to the consideration of "what art, (strictly speaking, the exhibition of medicine, or remedies powerfully efficacious in other diseases,) can do," in cases of insanity.

The application of medicine to mental disease must, of course, be directed, in the first instance, to the nature of the attack. We must inquire whether the brain is primarily or secondarily affected, and whether such affection is or is not connected with organic lesion. "The physical causes must be carefully separated from the moral, and due weight assigned to each.

"If, on accurate inquiry, the patient has been exposed to causes sufficient to produce inflammation of the brain, and if redness of the countenance, injection of the conjunctiva, and heat of the skin, very early in the disease, point out increased vascular action, with increased power, antiphlogistic remedies, with bloodletting, principally from the jugular vein, are to be employed; and in such cases, and probably only in such cases, mercury may be employed to affect the system; such cases will often recover." (P. 65.)

Bloodletting, however, must be cautiously employed in cases of insanity, in the treatment of which the same fatal practical fault is often committed as in cases of apoplexy. Many practitioners appear to imagine, if we judge from their practice, that, in madness and apoplexy, their principal duty consists in taking away large quantities of blood, rather than in determining whether the abstraction of any is really necessary. Dr. Haslam is quoted by Dr. Seymour upon this point: he informs us, that it is only in the very early stages of the disease (mania) that bloodletting is useful;" "and then he prefers cupping." According to his testimony, the appearance of the buffy coat on the blood, generally considered to justify such practice, has been found often absent in cases of lunacy where venesection has been used. "In more than two hundred patients, male and female, who were let blood by venesection, there were only six whose blood could be termed sizy. The experience of Pinel is decidedly against this practice."

For additional evidence upon the subject of bleeding in cases of mental derangement, Dr. Seymour refers to the medical officers of Mr. Warburton's house at Bethnal Green, containing four hundred patients.

"Messrs. Beverley and Phillips state the following as the result of their observations on this subject:

"The number of patients admitted with vascular excitement, requiring bloodletting, are very few indeed: we seldom or ever use the lancet in cases of excitement, if there is no evident effect upon the brain from increased arterial action, so as to lead us to fear an approaching attack of apoplexy or paralysis. The reason we do not use the lancet in cases without any such symptoms existing of disease going on in the brain, is, that we have done so in several instances, and the result was not favorable; the patient became reduced from the loss of blood, and the excitement was not abated; the powers of the constitution gave way, the tongue became typhoid, and the patient sank into a state of collapse, and died.

"As the result, then, of experience in cases of excitement, I presume that these arise from increased nervous energy, not depending on increased action of the heart and arteries, but on increased sensibility of the brain itself, and that bloodletting is not found useful." (P. 68.)

To allay the inordinately increased sensibility which appears so frequently to exist in insanity, and especially in that division termed dementia, various remedies have been employed. Cold is a common remedy in such cases; in the form of ice, as a shower-bath, and in a column of water, graduated according to the strength of the patient, and termed the "douche."

"Of ice to the head, not only in order to diminish vascular excitement, but also to produce a really sedative effect, a diminution of intensity of sensibility of the brain itself, I can speak with some confidence. In the epidemic typhous fever, which has recently raged among the lower classes in this town, and which has been of a marked and peculiar character, with scarcely any, if any, stage of excitement, the application of ice in the low maniacal delirium which accompanied it, was attended with the happiest effects, and in more than one case appeared to be the principal means of cure; the patient sleeping quietly for hours under an ice cap, who, previously to the use of the remedy, had passed the night in low muttering delirium, or in the constant endeavour to leave his bed. This remedy appeared to me to be useful exactly in proportion as the delirium assumed the maniacal character. The torpor produced by extreme cold may convey a tolerably accurate idea of the modification which the extreme sensibility of the brain may receive from this remedy. The effect which pouring cold water on the head, in cases of affection of the brain with increased vascular action, produces, may be illustrated by the following very remarkable case, which I attended with my friend Dr. Roupell, physician to the Seaman's Hospital.

"A young medical man was attacked with symptoms of inflammation of the brain, while pursuing with great ardour his professional studies; and being a medical man, he was, of all other persons, the most difficult to treat. Notwithstanding the intense pain in his head, and his quick and frequent pulse, he resisted remedies. Venesection he would not submit to; leeches he had an objection to; calomel he thought produced inflammation and ulceration of the mucous membrane of the intestinal canal: it was proposed to pour cold water from a pitcher over his head: the consequence was, diminution of pain, quiet sleep, and, in fact, so beneficial an effect, that the patient himself frequently called for a repetition of the remedy. I saw this patient several times, and the impression on my mind was, that to this remedy, and to this alone, he owed his life in that attack.

"The shower bath appears to have a very similar effect in cases of early excitement, and I am enabled to quote a case of its benefit in mania, from the testimony of the gentlemen to whom I have alluded.

"'We have found, in some cases, the shower bath of great service; but it appears to us, from the experience we have had of it, that it is more beneficial in cases of a very violent nature, with increased vascular excitement, as we have given it a trial in cases of various descriptions, and in some without the slightest benefit.'" (P. 70.)

In cases of melancholia, the warm bath has been found of service. Dr. Seymour was informed at Charenton, in France, and the Quaker's Retreat near York, that more marked advantage was derived from the employment of the warm bath in cases of melancholy, than from any known remedy in diseases of the mind; and Mr. Tuke states this opinion before the committee of the House of Commons. "The warm bath," he says, "is used more medicinally than the cold bath; and it has been observed that the warm bath has been found very beneficial, particularly in female cases." Dr. Guislain thinks favorably of the use of the warm bath, "especially in cases where lunatics refuse nourishment."

In chronic cases, both of mania and melancholy, producing discharges from the scalp by blisters, &c. has been recommended. Dr. Jenner believed that he had cured cases of insanity by producing pustules on the scalp. Dr. Seymour makes the following remarks:

"A case was related to me, on good authority, where mania, after continued fever, was cured by this means twice repeated; the symptoms of mania entirely disappearing with the second crop of pustules. I have, of course, endeavoured to inquire whether such beneficial effects are at all frequent. 'We have given this a trial,' say Messrs. Beverley and Phillips, 'more particularly with the tartar-emetic ointment, and with, we thought, some advantage, in a few cases; but we found, from the constant restlessness of the patient, erysipelas came on from the friction against the pillow, so that we were obliged to abandon it. We afterwards tried it in several cases over the biceps humeri muscle, with, I think, more benefit than on the head: these were cases of a very violent nature, stout robust habits, and who were, for the most part, subject to periodical attacks. The use of it had evidently the effect of making the paroxysms of much shorter duration. We have also used blisters to the inside of the thighs, and calves of the legs, with advantage.' The employment of tartar emetic internally may be directed on two principles: first, to diminish the action of the heart and arteries, where these are excited by keeping the patient in a constant state of nausea; secondly, as an emetic in melancholy, where full and repeated vomiting is of great service in producing a more rapid circulation in the vessels of the abdomen, and relieving the viscera, already gorged with blood. The former application of the remedy appears most applicable to mania; the second to monomania, or melancholy. 'Tartar emetic, in doses of one or two grains, given internally every hour, is worth noticing in patients who are subject to violent paroxysms, particularly those who have increased vascular excitement, with great restlessness. The patient generally complains of nausea without vomiting, becomes languid and quiet, rests better, appetite improves, and in a few days is trusted out of confinement.'" (P. 76.)

In addition to these means of diminishing sensibility and irritability, sedative medicines have been tried, and especially opium. The opinions respecting the use of this agent in mania are various, but Dr. S. thinks may be easily explained.

"Where vascular excitement exists, together with increased sensibility of the brain, the restlessness is increased by the administration of opium; where perverted perception arises from disorder of the functions of the abdominal viscera, the constipating effect of this medicine, and the manner in which it acts to diminish all the secretions, obviously renders it hurtful; and it must be added, that in some constitutions it is poison. Hence it is far from generally useful even in the cases in which it is indicated; and it is often the case that physicians neglect medicines (after a time) which do not entirely fulfil their expectations, so occasionally this medicine has been unduly estimated, and immediately afterwards as improperly neglected. In some instances mistaken pathology has lent its aid to undervalue the medicine, and one is forced occasionally to listen to theoretic doubts about its efficacy in cases of great and urgent irritability, from the prevalent opinion that all disease arises from inflammation, acute or subacute, and the corresponding error that opium necessarily occasions congestion in the brain. There is no question that, where opium is not contra-indicated from peculiarity in the individual, or the presence of another disease to which it is ill adapted, it is of all others the remedy to diminish the sensibility and irritability of the system, to make the wretched forget his grief, the ruined his poverty, and even the criminal the mental retribution of his wickedness: and in the same way it often removes from the imagination of the maniac his supposed iniquities. Still partly the real, and partly the theoretical objections to this remedy, have caused physicians to seek for some substitute from the class of sedatives which would exhaust that increased sensibility which magnifies a hundred fold the objects presented to it." (P. 78.)

The acetate of morphia is thought to be very efficacious in mania; and cases are related in the work to prove its good effects. Henbane and belladonna have also been frequently tried: of the latter, Dr. Seymour thinks highly.

"I have used it extensively in St. George's Hospital; and, as far as my practice would permit, in private life, and often with the most marked benefit. The following is one of the cases in which its alleviating powers were best observed: A general officer was attacked, about fourteen years ago, with the painful affection of the second branch of the fifth pair of nerves, tic doloureux. He underwent various remedies from the recommendation of the most celebrated physicians in London. The disease yielded to very large doses of belladonna, and it never reappeared for ten years. At the expiration of this time he had an apoplectic seizure, which left paralysis of the right side, very imperfect condition of speech, and frequent convulsive and painful affections of the nerves, sometimes choking, and at other times startings of the nerves of the affected limb, with occasionally the return of the tic doloureux. It is impossible to conceive more acute pain than is experienced by the patient at these times. He always (and I have witnessed at least ten such paroxysms) finds relief in the extract of belladonna. Half a grain is administered every four hours; and always when the third dose has had time to operate, the pains and spasmodic twitches disappear. It might be supposed that the pain terminated of itself, or that the spasmodic affection ceased naturally at this time; but, distressed at the weak state in which the belladonna leaves him, the patient has once or twice refused to take it, and he has suffered intense pain much longer than he otherwise would have done, being obliged to resort to the remedy at last.

"Greding speaks highly of this medicine in epilepsy; and in one or two cases in the hospital, it has appeared to have rendered the intervals much longer, but I cannot lay claim to a single cure. It appears to me well worth the trial of physicians in mania, especially that which arises from moral causes, and is attended with pain and increased sensibility of the brain." (P. 85.)

Hydrocyanic acid has been rarely tried in this country in cases of insanity. Arsenic "appears to alter the sensibility and irritability of the brain, and has long been known as very efficacious in intermittent diseases." Dr. Seymour has used it with advantage in some instances which were nearly allied to mania.

"A woman, æt. forty, (servant to a lady in the Regent's Park,) was attacked with constant pain in the head, confusion, and giddiness, attended at times with loud noises, which she described like breaking stones: at other times images presented themselves to her mind, and her nights were entirely sleepless; pulse quick and weak. This condition continued for months, during which time I employed every means in my power for her relief: at length I resorted to the employment of arsenic. The patient took ten minims of the liquor arsenicalis twice daily, subsequently increased to the same quantity three times in the day. In about a week very great improvement had taken place; the pains had diminished, and the nights were tranquil. In a fortnight her ailments entirely left her.

"The next was the case of John Graham, æt. thirty, admitted into St George's Hospital, under my care, November 24th, 1830. The report, on admission, was as follows: Ill since June last, with pain in the forehead, diverging to each side, very excruciating; and reported to feel as if the head was forced or burst open. The pains are worse at night, so as entirely to prevent sleep. The pupils are dilated, and the conjunctivæ injected with blood; sense of smell increased in acuteness; bowels constipated; pulse 100, very weak; skin cold.

"The bowels were ordered to be kept open, and sedative and antispasmodic medicines were employed without effect: on the 6th of December, he was ordered five minims of liquor arsenicalis twice in the day.

"11th. The pains were reported greatly relieved.

"13th. The pupils contracted naturally; the pain and pressure in the head nearly gone, except when he lies down.

"The medicine increased to five minims thrice daily: from this time the pains entirely disappeared. He remained in the house a fortnight, to see if any relapse took place, when he was dismissed free from ailment." (P. 88.)

In some cases, particularly with female patients, when the symptoms resemble hysteria, camphor has been often and beneficially used: Dr. Perfect relates the following example.

"Mrs. S. B., a married woman, became melancholic: her complexion was pale, eyes red, tongue dry, pulse small, hard, and irregular. After bleeding and vomiting, Dr. Perfect ordered two scruples of camphor to be taken morning and evening. An eruption came out, after this treatment, all over the body. The catamenia returned: nitre was added to the camphor, and the patient recovered completely." (P. 90.)

Purgatives are often useful; and the oil of croton may be frequently preferred, as it may always be given even to unruly patients. In cases of hysteria connected with epilepsy, oil of turpentine has been recommended by many good authorities.

"The belief that cases of insanity always depend on organic causes, or a state of acute or subacute inflammation of the brain, has not only led to the erroneous treatment of many of these forms of disease by venesection, but has suggested an antiphlogistic diet. We have, I think, the best evidence, from the greatest experience in France and England, of the evil consequent on such practice. M. Pinel draws a terrible picture of the afflicting consequences of the short allowance on which the patients of the Bicêtre were placed in 1796; and it has been seen that a full diet, and even considerable quantities of stimulants, are often productive of the utmost advantage in what are termed high cases. In the cases arising from childbed, or during nursing, Dr. Gooch has very clearly demonstrated that these occur either in consequence of, or during an exhausting process, and are relieved by tonic and anti-spasmodic medicines and restorative diet." (P. 93.)

Our analysis of Dr. Seymour's brief but valuable work is now completed: from its perusal we have derived much pleasure and instruction.

---

*Elements of Chemistry, including the recent Discoveries and Doctrines of the Science.* By Edward Turner, M.D., F.R.S. L. & E., SEC. G.S., Professor of Chemistry in the University of London, &c. *Third Edition, enlarged and carefully revised.*—8vo. pp. 908. John Taylor, London.

So many are the works which monthly issue from the press, that, giving (as we are bound to do,) our earliest and chief attention to those most strictly medical, we find ourselves occasionally post-poning, for a longer period than we could wish, our notices of some, which, although important in themselves, are only collaterally important: and thus it is with Dr. Turner's "Elements of Chemistry," that now have reached a third edition within the short space of four years. We, however, the less regret our seeming neglect in this case, as it has enabled us to speak in terms of much less qualified commendation than we could hitherto have done of a work that, from the first, we have highly valued; for nearly two hundred pages have been added and rewritten, and many of the parts we then thought meagre have been enlarged: still we must confess that there are some remaining, especially in the departments of organic chemistry, which we would fain see further amplified.

The introductory chapters, dedicated to a brief consideration of Heat, Light, Electricity, and Galvanism, are well written, and we recommend them to the attentive perusal of the student: but we admit that our favorite chapter is that devoted to the "Laws of Combination;" and although we prefer the title "Theory of Definite Proportions," to that of the "Atomic Theory," yet, when the facts are so lucidly arranged and clearly stated as we find them here, we are not disposed to quarrel with a name. It will scarcely, however, admit of extracts being made, for the matters it relates to are purely elementary, and its value consists in the succinct yet perspicuous mode in which the results of much laborious investigation has been stated, and the tact with which many important truths have been separated from much unimportant hypothesis. This, indeed, seems to be Dr. Turner's forte; and, as an illustration, we may select the notice of the late Dr. Wells' theory of Dew.

"The most copious deposition of dew takes place when the weather is clear and serene; and the substances that are covered with it are always colder than the contiguous strata of air, or than those bodies on which dew is not deposited. In fact, dew is a deposition of water previously existing in the air as vapour, and which loses its gaseous form only in consequence of being chilled by contact with colder bodies. In speculating, therefore, about the cause of this interesting and important phenomenon, the chief object is to discover the principle by which the reduction of temperature is effected The explanation proposed by Dr. Wells, and now almost universally adopted, is founded on the theory of M. Prevost. If it be admitted that bodies radiate at all times, their temperature can remain stationary only by their receiving from surrounding objects as many rays as they emit; and should a substance be so situated that its own radiation may continue uninterruptedly without an equivalent being returned to it, its temperature must necessarily fall. Such is believed to be the condition of the ground in a calm starlight evening. The calorific rays which are then emitted by substances on the surface of the earth, are dispersed through free space and lost; nothing is present in the atmosphere to exchange rays with them, and their temperature consequently diminishes. If, on the contrary, the weather is cloudy, the radiant caloric proceeding from the earth is intercepted by the clouds, an interchange is established, and the ground retains nearly, if not quite, the same temperature as the adjacent portions of air.

"All the facts hitherto observed concerning the formation of dew, tend to confirm this explanation. It is found that dew is deposited sparingly, or not at all, in cloudy weather; that all circumstances which promote free radiation are favorable to the formation of dew; that good radiators of caloric, such as grass, wood, the leaves of plants, and filamentous substances in general, reduce their temperature, in favorable states of the weather, to an extent of ten, twelve, or even fifteen, degrees below that of the circumambient air; and that while these are drenched with dew, pieces of polished metal, smoothed stones, and other imperfect radiators, are barely moistened, and are nearly as warm as the air in their vicinity." (P. 21.)

The curious experiments of Mr. Pearsall on the Phosphorescence of Minerals, we think, must have been published some time before this last edition could have gone to the press: if so, they should have been noticed, and we have therefore felt disappointed that a subject hitherto so obscure, and on which they have thrown so much light, has not led to their incorporation here.

The brilliant electro-chemical discoveries of Davy need no mention now; but his experiments on the protective influence of galvanic contact against the corrosive action of sea water on the copper sheathing of vessels, which, although philosophically successful, practically failed, by giving rise to another inconvenience as great as that they were designed to remedy, and the failure of which not improbably shortened his life, cannot well be passed over, and we therefore extract the following paragraphs.

"It has been long known that the copper sheathing of vessels oxidizes very readily in sea water, and consequently wastes with such rapidity as to require frequent renewal. Sir H. Davy observed that the copper derived its oxygen from atmospheric air dissolved in the water, and that the oxide of copper then took muriatic acid from the soda and magnesia, forming with it a submuriate of the oxide of copper. Now, if the copper did not oxidize, it could not combine with muriatic acid; and, according to Sir H. Davy, it only combines with oxygen, because, by contact with that body, it is rendered positively electrical. If, therefore, the copper could by any means be made negative, then the copper and oxygen would have no tendency to unite. The object, then, was to render copper permanently negative. Now this is done by bringing copper in contact with zinc or iron; for the former then becomes negative, and the latter positive.

"Acting on this idea, it was found that the oxidation of the copper may be completely prevented. A piece of zinc as large as a pea, or the head of a small round nail, was found fully adequate to preserve forty or fifty square inches of copper; and this where-ever it was placed, whether at the top, bottom, or middle of the sheet of copper, or under whatever form it was used: and when the connexion between different pieces of copper was completed by wires, or thin filaments of the fortieth and fiftieth of an inch in diameter, the effect was the same; every side, every surface, every particle of the copper remained bright, whilst the iron or the zinc was slowly corroded. Sheets of copper defended by 1-40th to 1-1000th part of their surface of zinc, malleable and cast iron, were exposed during many weeks to the flow of the tide in Portsmouth harbour, and their weight ascertained before and after the experiment. When the metallic protector was from 1-40th to 1-150th there was no corrosion nor decay of the copper; with smaller quantities, such as 1-200th to 1-460th, the copper underwent a loss of weight which was greater in proportion as the protector was smaller; and, as a proof of the universality of the principle, it was found that even 1-1000th part of cast iron saved a certain proportion of the copper. (*Phil. Trans.* for 1824.)

"Unhappily for the application of this principle in practice, it is found that, unless a certain degree of corrosion takes place in the copper, its surface becomes foul from the adhesion of seaweeds and shellfish. The oxide and submuriate of copper, formed when the sheathing is unprotected, is probably injurious to these plants and animals, and thus preserves the copper free from foreign bodies. It appears also that, in vessels whose sheathing is protected from corrosion, the negatively electric copper attracts the positively electric bodies, such as magnesia and lime, dissolved in sea water; and that these earths then form a nidus for the adhesion of other matters. It is hoped that, by duly adjusting the proportion of iron and copper, a certain degree of corrosion may be allowed to occur, sufficient to prevent the adhesion of foreign bodies, and yet materially to retard the waste of the copper; but the attempts to accomplish so desirable an object have not yet been altogether successful." (P. 128.)

The remarks on the constitution and chemical similarity of Atmospheric Air, in all places and under all circumstances, shew that chemistry has not effected all that probably it will hereafter do; for we find the same confession here which has been repeated in such works time almost immemorial, (i. e. as long as this subject has been noticed,) viz. that

"Air collected at the summit of the highest mountains, such as Mont Blanc and Chimborazo, contains the same proportion of oxygen as that of the lowest valleys. The air of Egypt was found by Berthollet to be similar to that of France. The air which Gay-Lussac brought from an altitude of 21,735 feet above the earth, had the same composition as that collected at a short distance from its surface. Even the miasmata of marshes, and the effluvia of infected places, owe their noxious qualities to some principle of too subtile a nature to be detected by chemical means, and not to a deficiency of oxygen. Seguin examined the infectious atmosphere of an hospital, the odour of which was almost intolerable, and could discover no appreciable deficiency of oxygen, or other peculiarity of composition." (P. 225.)

It would have been well if the rationale of the action of plants on atmospheric air had been further explained than we find it in the following passage, which seems to imply that two effects proceed from the same cause: whereas, it was proved by Mr. Burnett, in an essay on this subject, published in the first number of the Royal Institution Journal, that the two opposite effects are the results of two different functions, one of which he considers analogous to the digestion, the other to the respiration of animals; the latter of which is always going on, both by night and by day, in health and in disease; the other only occasional, and only very effective in healthy and growing plants; which is a doctrine very different from that inculcated here.

"There is still one circumstance for consideration respecting the atmosphere. Since oxygen is necessary to combustion, to the respiration of animals, and to various other natural operations, by all of which that gas is withdrawn from the air, it is obvious that its quantity would gradually diminish, unless the tendency of those causes were counteracted by some compensating process. To all appearance, there does exist some source of compensation; for chemists have not hitherto noticed any change in the constitution of the atmosphere. The only source by which oxygen is known to be supplied, is by the action of growing vegetables. A healthy plant absorbs carbonic acid during the day, appropriates the carbonaceous part of that gas to its own wants, and evolves the oxygen with which it was combined. During the night, indeed, an opposite effect is produced: oxygen gas then disappears, and carbonic acid is eliminated; but it follows, from the experiments of Priestley and Davy, that plants, during twenty-four hours, yield more oxygen than they consume. Whether living vegetables make a full compensation for the oxygen removed from the air by the processes above mentioned, is uncertain. From the great extent of the atmosphere, and the continual agitation to which its different parts are subject by the action of winds, the effects of any deteriorating process would be very gradual, and a change in the proportion of its elements could be perceived only by observations made at very distant intervals. (P. 228.)

It may, perhaps, be not amiss to mention, that Brande has made a calculation by which he endeavours to prove that comparing the proportion of atmospheric air deteriorated by combustion, by the respiration of plants, of animals, and of about eight hundred millions of human beings, with the entire bulk of the atmosphere, the change would be so small as to be inappreciable by experiments carried on within the periods since pneumatic chemistry has flourished.

It is evident that this volume has been chiefly written, as indeed it professes to be, for the use of students attending chemical lectures, and as such, and for such readers, it would doubtless be found a very useful text-book: but this is not all, for we can conscientiously recommend it as a valuable work for the perusal of all who have attended, as well as those who are now attending, courses of practical demonstrations: it will bring many things to remembrance which, perhaps, have been half forgotten, and explain others which, in the rapid progress of modern science, a few years since were utterly unknown. Doubtless the accomplished chemist will find many points too cursorily treated, and others (as is the case with that very curious body Oxalamide,) entirely omitted; but so much has really been compressed into nine hundred pages, that we are perhaps unreasonable in desiring more; and, possibly, the parts to which we have and might refer as less complete than we could wish them, had passed the author's supervision before the accounts of some of the discoveries, by which they might have been made more perfect, had reached his notice.

It is, indeed, the bane of books on the natural sciences, and yet one of the most valued privileges of philosophy, that knowledge so rapidly increases that a work can scarcely be got through the press, even aided by steam, before some parts of it have become obsolete.

*A History of British Animals; exhibiting the Descriptive Characters and Systematic Arrangement of the Genera and Species of Quadrupeds, Birds, Reptiles, Fishes, Mollusca, and Radiata, of the United Kingdom: including the indigenous, extirpated, and extinct Kinds, together with periodical and occasional Visitants.* By John Fleming, d.d., f.r.s.e., m.w.s., &c.—8vo. pp. 565. Bell and Bradfute, Edinburgh; Duncan, London.

This is a very creditable work, and one that we have no hesitation in recommending as a very convenient manual to the young naturalist; for it is arranged on a much more philosophic principle, and embodies more of the modern doctrines and discoveries than we find in any other work of equal size, and of a similar date. The including the extinct and extirpated species, perhaps, has won our especial favour; for we have often regretted that, until lately, so little attention has comparatively here been paid to fossil organic remains, in which this country is very rich; while, in France, Cuvier and his followers have been reaping well-earned harvests of renown.

We suppose that the peculiar habits and modes of propagation of the Planeriæ were unknown to our author when this volume was written: indeed, many of the researches are quite of the present moment, and, as they may not be in general known, we subjoin an account of some experiments we ourselves have witnessed.

This genus (Planeria) is allied to the leech, and there are four or more species indigenous to our ponds and ditches, viz. the P. torva, lactea, felina, &c. The species which inhabit stagnant water are viviparous and oviparous; those living in running water are viviparous only: but it is not merely this circumstance which gives an interest to these animals, but the mode in which the reproduction occurs; e. g. if the head of a planeria be split, so as to divide the eyes, a new portion will be produced between the sections, upon which will be formed one or two more eyes; or, if the section be made very deep, each old half will form for itself a new half, so that the animal will have two heads, each bearing two eyes; and, if these be again split, they will each form other halves, so that the heads may be multiplied at pleasure. If the head or heads be cut off below the bifurcation, then a new single head will be formed from the body, and the multiceps be restored to its pristine state. If the longitudinal section be continued further down the body, two entire animals will be formed by the two halves, and only be united by the single tail; and so unharmoniously do these siamoid planeriæ seem to live, that, after a short struggle, they tear themselves asunder. If the body of a planeria be cut into two pieces, the head will swim away, scarcely appearing to miss its tail, and a new posterior half will shortly be produced; but the tail, sinking to the bottom of the vessel, remains torpid for two or three days; after which time it again becomes invested with a head, as perfect as the first. Indeed, so far may these divisions be carried artificially, that six or eight entire individuals may be made by cutting one into as many pieces. Moreover, these animals naturally, and as it were at pleasure, throw off pieces of their bodies as they swim along, each of which in a short time becomes as perfect as its parent.

This viviparous generation solely goes on in permanent pools and running streams; but in those places in which the water occasionally dries up, the planeria become oviparous. Such persons as wish to verify these curious phenomena, which so closely approximate the propagating powers of animals and plants, may procure plenty of planeria in the neighbourhood of the Red House, Battersea fields.

---

*The Universal System;* by GEORGE FIELD. (*Pamphleteer*, Nos. XVII. XXIV. XXIX. XXXIII. XXXVI.)

*Horæ Entomologicæ;* by WILLIAM SHARP MACLEAY.

*The Philosophy of Zoology;* by JOHN FLEMING, D.D.

*Illustrations of Nature;* by GILBERT T. BURNETT. (*Journal of Science*, N. S., Nos. VI. VIII. X. XII. XIII.)

WE place these works as a heading to our pages, not so much for the purpose of specifically reviewing either of them, as with the intent of recalling to the notice of our physical readers the varied numerical schemes which the above-named gentlemen have been labouring for several years past to introduce, and by which they argue that the theory of nature and of knowledge would be much simplified. Each defends his system with considerable ingenuity, and apparently with much success, until his opponent cometh to put him to the test: which withstands the ordeal best, it becomes not us to say, when we merely wish to expound their several doctrines, and that in the most cursory manner.

Hitherto we have been content to follow our studies in the paths our progenitors tracked out, and to associate the productions of nature in three kingdoms, as our forefathers have done before us; but now we are told, and with much seeming plausibility, that such old arrangements (if arrangements they could, in many points, be called,) are fundamentally erroneous, and that neither nature nor knowledge have been demarcated correctly.

It seems that all agree we have been hitherto in the wrong; but, unfortunately, a similar agreement is not found as to which course would ensure our journeying right in future: for one author proposes to simplify systematic arrangements by constantly distributing all physical existence into dichotomies, some prefer a triple distribution, some a quintuple, and some a septuple division; thus giving rise to the binary, ternary, quinary, and septenary systems.

The first has simplicity in principle to recommend it; but in practice it is tedious in its process, without the promise of any compensating advantage; it is confessedly an artificial scheme, with machinery more cumbersome than those which aim at an approximation to a natural method.

Messrs. FIELD and MACLEAY, KIRBY and SPENCE, and others, appear to assume a certain number, be it three, or five, or seven, as essential to their plans, which numbers they respectively consider as consecrated by nature, who, according to their views, has formed plants, animals, &c. in triple, quintuple, or septuple groups; which groups it should be the object of the naturalists to find out: it, however, not improbably may be shewn that such groups are no more formed by nature, than the statue was naturally formed in the rock; and often, we believe, if the naturalist would find them, he must do as the artist does to find his figure, he must *make* them.

Mr. BURNETT professes, on the other hand, not to assume any particular number, although he allows that his researches incline him to believe that a trine distribution very generally prevails; his object being rather to indicate the connexions than to separate the provinces of nature and of knowledge; and, to illustrate his views, he adopts intersecting circles as symbols; while the quinary philosophers prefer osculant rings, and the author of the Universal System has aptly chosen the triangle, which, in its analysis, is osculant likewise.

The essays of Mr. FIELD are evidently the result of much abstract reflection; those of Mr. Burnett of much physical research; while the Horæ Entomologicæ, much the most celebrated and generally known, would seem to be the amplification to nature generally of a principle deduced from the examination of a small group of insects.

Without copious extracts from these several essays, which, for the most part, are written with sententious brevity, we could not hope to convey a clear conception of the objects they severally attempt to achieve; and, as our purpose has been not to offer an analysis, but rather to direct the attention of our physical readers to the original papers, which, from the circumstances of the form in which two series have been published, and the destruction by fire of almost the entire impression of the Horæ Entomologicæ, are much less read, and much less known, than, in our opinion, they deserve to be. Our duty is now performed, and we shall conclude with a recommendation to such persons as may in future find it expedient to adopt the ideas and principles of these several theorists, to acknowledge the sources whence they are derived, and not, by badly transposing, think to make them their own.

